# 0990. Role of amplitude and rate of deformation in ventilator-induced lung injury

**DOI:** 10.1186/2197-425X-2-S1-P75

**Published:** 2014-09-26

**Authors:** A Protti, DT Andreis, M Milesi, P Pugni, F Nicosia, T Maraffi, V Melis, M Monti, E Votta, L Gattinoni

**Affiliations:** Fondazione IRCCS Ca' Granda - Ospedale Maggiore Policlinico, Milan, Italy; Università degli Studi di Milano, Milan, Italy; Politecnico di Milano, Milan, Italy

## Introduction

Increasing both Tidal Volume (V_T_) (*amplitude* of lung deformation) and Inspiratory Flow (V') (*rate* of lung deformation) augments incidence of Ventilator-Induced Lung Injury (VILI) [1].

## Objectives

To clarify whether increasing V' at constant V_T_ augments incidence of VILI.

## Methods

Twenty-eight healthy piglets were mechanically ventilated for up to 54 hours. Each animal was assigned to one of three groups of V_T_ (300-400 ml; 500-600 ml; 750 ml) and one of two groups of V'. Lower and higher V' were obtained by setting inspiratory-to-expiratory time ratio as high as 1:2 or as low as 1:9. Respiratory rate was always 15 breaths per minute. Interplay between V_T_ and V' was assessed at the beginning of the study as airway pressure-volume loop area (or dynamic respiratory system hysteresis). VILI was defined as pulmonary oedema (lung weight gain ≥10% across the study period).

## Results

Main findings are reported in Table 1.

**Table 1:** Inspiratory flow and incidence of VILI

	V_T_ 300-400 ml		V_T_ 500-600 ml		V_T_ 750 ml	
	**Lower V´**	**Higher V´**	**Lower V´**	**Higher V´**	**Lower V´**	**Higher V´**
Tidal volume (ml)	338±48	335±42	530±27	520±27	750±0	750±0
Inspiratory flow (ml/sec)	272±36	838±105*	398±21	1278±38*	600±84	1242±95*
Hysteresis (ml*cmH_2_O)	6260±2236	12938±3356*	11101±4508	34126±4508*	18415±3520	46915±7954*
Incidence of VILI	0/4	1/5	0/5	4/5*	2/5	4/4

Data are presented as mean±standard deviation. * p< 0.05 *vs.* Lower V' within the same V_T_ group (Student's *t*, Mann-Whitney Rank Sum or Fisher's exact tests). Hysteresis was associated with incidence of VILI (R=0.68, p< 0.0001) (Spearman Rank Order Correlation).

## Conclusions

Increasing V' (*rate* of lung deformation) while maintaining V_T_ (*amplitude* of lung deformation) constant augments incidence of VILI. Further studies are needed to clarify whether dynamic respiratory system hysteresis is an independent predictor of VILI.

## References

[1] Protti A (2011). Lung stress and strain during mechanical ventilation: any safe threshold?. Am J Respir Crit Care Med.

